# Advancements in Endoscopic Optical Coherence Tomography for Urothelial Carcinoma: Research Progress and Clinical Applications

**DOI:** 10.2174/0115734056434411260224060501

**Published:** 2026-03-18

**Authors:** Yongkun Zhu, Qiang Hu, Rui Zhang, Guangyuan Zhang, Ming Chen

**Affiliations:** 1 Urology Department, Zhongda Hospital, Southeast University, Nanjing, Jiangsu, China; 2 Urology Department, Zhongda Hospital, School of Medicine, Southeast University, Nanjing, Jiangsu, China; 3 Ophthalmology Department, Zhongda Hospital, Southeast University, Nanjing, Jiangsu, China

**Keywords:** Optical coherence tomography, Urothelial carcinoma, Cancer staging, Treatment monitoring, Intraoperative guidance, Magnetic resonance imaging, Narrow-band imaging

## Abstract

**Background:**

This review summarizes recent advances in endoscopic optical coherence tomography for urothelial carcinoma, focusing on technical features, clinical applications, and future directions.

**Materials and Methods:**

This is a narrative review based on a literature review of databases such as PubMed, Embase, Web of Science, and the Cochrane Library, from January 2000 to March 2025. OCT imaging diagnosed with urothelial carcinoma. Studies meeting the following inclusion criteria are eligible, including reports describing the technical aspects or clinical applications (such as diagnosis, staging, intraoperative guidance, or postoperative monitoring) of OCT imaging in the care of patients with urothelial carcinoma. The results of the study have been categorised.

**Results:**

OCT demonstrated high-resolution imaging and real-time capabilities, enabling differentiation of malignant from normal urothelial tissues. Clinical studies confirmed its value in tumor staging and intraoperative guidance, with strong concordance to histopathology. OCT improved diagnostic sensitivity and specificity when combined with conventional cystoscopy and showed potential in monitoring treatment response and detecting recurrence.

**Discussion:**

OCT offers a non-invasive, precise imaging modality with significant advantages over traditional diagnostic methods. However, limitations remain, including restricted imaging depth and variability across clinical protocols. Standardization and broader clinical validation are needed to ensure consistent adoption.

**Conclusion:**

Endoscopic OCT represents a transformative tool for UC management. With further technological innovations and standardized protocols, it is expected to enhance early detection, intraoperative guidance, and long-term patient outcomes.

## 1 INTRODUCTION

Urothelial Carcinoma (UC) is one of the most common malignancies of the urinary system and can occur in any region lined by urothelium, including the renal pelvis, ureters, bladder, urethra, and urachus. Early diagnosis is crucial for improving patient survival rates [1, 2]. Traditional diagnostic methods primarily rely on imaging techniques and tissue biopsies. While these approaches are widely used in clinical practice, they have certain limitations. For instance, imaging techniques may fail to accurately distinguish between benign and malignant lesions, while tissue biopsy is invasive and carries the risk of complications [3].

Endoscopic Optical Coherence Tomography (OCT) is an emerging imaging technology that offers advantages such as non-invasiveness, high resolution, and real-time imaging. In recent years, OCT has made significant progress in the early diagnosis of tumors. Studies have shown that OCT enhances tissue contrast, effectively differentiating normal from malignant renal tissue [4]. Additionally, OCT enables high-resolution subsurface characterization of suspicious lesions, providing microstructural and cellular morphological details that cannot be obtained through conventional macroscopic imaging. This capability enhances the sensitivity and specificity of bladder cancer diagnosis [5].

With its high resolution, real-time imaging, and expanding clinical use, endoscopic OCT has the potential to refine the diagnosis and management of UC in the future [6].

## 2 MATERIALS AND METHODS

### 2.1 Literature Search Strategy

This narrative review was written following a systematic literature search with PubMed, Embase, Web of Science, and the Cochrane Library (January 2000 - March 2025). We applied a structured search strategy using keywords (*e.g*., Optical coherence tomography, OCT, endoscopic, cystoscopy, urothelial carcinoma, Bladder cancer) and their synonyms, and controlled vocabulary terms (MeSH/Emtree) where applicable. To facilitate complete data, the references of important articles were also included to find more relevant studies. The literature data were used as a basis for an overview of the technological advances and clinical applications of endoscopic OCT in urothelial carcinoma.

### 2.2 Inclusion and Exclusion Criteria

This review focuses on the studies dealing with endoscopic OCT applications on urothelial carcinoma. The system design and technical developments, imaging features, diagnostic or staging accuracy, intraoperative guidance, or monitoring role may be interesting topics. Original papers or clinically oriented technical reports were prioritised and excluded only if the discussion focused on non-endoscopic OCT techniques, non-urothelial malignancies, or applications not directly applicable to urothelial carcinoma.

### 2.3 Literature Screening and Data Extraction

Retrieved records were screened for individual studies and selected according to the aims of the narrative review. Study design and setting, composition of OCT systems (resolution and type probe), patient or sample features, reference stand-ards, and main findings with respect to clinical utility were qualitatively summarised. This synthesis aimed to support a thematic analysis rather than a formal quantitative aggregation.

### 2.4 Quality Assessment

Since the OCT technology, study protocols, and outcomes are heterogeneous, we did not perform a systematic risk-of-bias evaluation or a PRISMA-guided evaluation for the studies. We comprehensively assessed each study's design, sample size, the reference standard, and reporting readability in understanding the strengths and limitations of available evidence.

### 2.5 Data Synthesis

The results of the study were summarised through qualitative methods, and the study was organised into thematic sections. In this study, the topics addressed include (i) fundamental principles and technological advancements in endoscopic OCT, (ii) diagnostic imaging features of urothelial carcinoma, (iii) clinical applications such as diagnosis, staging, and intraoperative guidance, and (iv) current limitations and future trends. The performance criteria for the reported studies are presented as ranges, and the study findings are discussed based on the background analysis due to differences in devices, populations, and references used. This approach was chosen specifically to avoid the use of pooled estimates, thereby preserving the distinct characteristics of each study.

## 3 RESEARCH PROGRESS AND CLINICAL APPLICATIONS OF ENDOSCOPIC OPTICAL COHERENCE TOMOGRAPHY IN UROTHELIAL CARCINOMA

### 3.1 Overview of Endoscopic Optical Coherence Tomography Technology

#### 3.1.1 Principles and Working Mechanism

Endoscopic OCT is an optical interference-based imaging technology that generates high-resolution 3D images of internal tissues. It employs a low-coherence light source to emit a beam split into sample and reference pathways [7]. By analyzing phase differences between reflected signals from the sample and reference beams, OCT measures depth-resolved tissue reflections, producing micron-level resolution cross-sectional images, significantly outperforming traditional ultrasound and X-ray in spatial detail [8]. Figure **1** schematically illustrates a typical endoscopic OCT system. A fiber coupler directs light from the low-coherence source into a reference arm terminating in a mirror and a sample arm connected to an endoscopic OCT probe inserted into the urinary tract lumen. Backscattered light from the tissue and reflected light from the reference mirror recombine at the coupler to form an interferometric signal, which is then detected and processed by the OCT engine to reconstruct depth-resolved cross-sectional images.

#### 3.1.2 Advantages Compared to Traditional Imaging Techniques

As a non-invasive imaging technology, OCT offers several advantages over conventional modalities. Firstly, OCT uses a low-coherence light source and interferometry to provide real-time, non-contact, high-resolution cross-sectional images of biological tissues [9]. Its axial resolution can reach 1-2 μm, far exceeding that of high-resolution ultrasound and approaching microscopic resolution, making it particularly suitable for visualizing fine mucosal and submucosal structures [10, 11]. Secondly, OCT offers high spatial resolution, reported to be up to 10-fold higher than intravascular ultrasound, allowing detailed depiction of tissue microarchitecture and device-tissue interfaces [12]. Moreover, OCT can penetrate 2-3 mm into optically scattering tissues, enabling *in vivo* histology-like assessment without tissue removal or preparation, which is why it is often referred to as “optical biopsy” [13, 14]. The rapid imaging speed permits acquisition of large datasets within seconds, supporting dynamic clinical monitoring and timely assessment of lesion changes [15, 16]. Additionally, OCT is multifunctional, non-ionizing, and relatively cost-effective, with broad applications across medicine, particularly in ophthalmology and dentistry, and it can generate multidimensional *in vivo* datasets for combined structural and functional imaging [17].

Compared with ultrasound (US) and Computed Tomography (CT), OCT therefore provides more detailed local information on the urothelial wall and superficial layers, whereas conventional imaging remains superior for whole-organ and extraluminal assessment; these modalities are best regarded as complementary rather than competing techniques. The specific features and clinical applications of OCT are summarized in Table **1**.

Beyond OCT, several other advanced endoscopic imaging techniques have been introduced for UC, including Narrow-Band Imaging (NBI), photodynamic or fluorescence cystoscopy, and Confocal Laser Endomicroscopy (CLE). NBI and fluorescence cystoscopy enhance visualization of super-ficial and flat lesions by improving mucosal and vascular contrast or highlighting areas of increased photosensitizer uptake, but they do not provide depth-resolved structural information [18, 19]. CLE offers cellular-level “optical biopsies” with very high resolution in a small field of view and limited penetration [20]. In contrast, OCT depicts depth-resolved cross-sectional architecture over a relatively large area.

#### 3.1.3 Current Status of Clinical Applications

Currently, OCT technology has been widely applied in various clinical fields, especially in ophthalmology, cardiovascular diseases, and tumor detection. In ophthalmology, OCT is extensively used for the early diagnosis and monitoring of retinal diseases, such as macular degeneration and glaucoma [21, 22]. In the cardiovascular field, OCT provides clear imaging of plaques and thrombi within blood vessels, offering critical information for interventional treatments [23, 24]. In tumor detection, OCT applications are also increasing, enabling physicians to observe the microstructure of tumor tissues in real-time under endoscopic guidance, assisting in pathological diagnoses [25]. Despite the promising clinical potential of OCT, challenges remain, such as limited imaging depth and image quality degradation due to tissue scattering [26]. Therefore, future research will focus on enhancing imaging depth and optimizing imaging algorithms to further expand the clinical applications of OCT.

### 3.2 Applications of OCT in Early Diagnosis of Urothelial Carcinoma

#### 3.2.1 Identification of Lesion Features

As an emerging imaging technology, OCT has gradually been applied to the early diagnosis of UC. OCT provides high-resolution tissue imaging, assisting physicians in identifying tumor lesion characteristics. Studies have shown that OCT can clearly visualize microscopic changes in urothelial structures, such as thickening of the cell layers, disorganized cell arrangement, and basal membrane disruption, early indicators of UC [27]. For instance, through OCT imaging, physicians can observe optical features of tumor tissues, such as changes in reflectance intensity and variations in tissue optical density. These features may differ across various types of UC. By comparing OCT images of normal and diseased tissues, clinicians can more accurately identify cancerous regions, providing a reliable basis for subsequent treatment decisions [28].

#### 3.2.2 Quantitative Analysis and Tumor Staging

In addition to its role in qualitative diagnosis, OCT can also assist in tumor staging through quantitative analysis. Studies have indicated that OCT can help determine tumor stage by measuring the thickness, volume, and relationship of the tumor to surrounding tissues [29]. For example, OCT can quantify tumor growth patterns, including invasion depth and boundary characteristics, which are crucial for assessing tumor aggressiveness and prognosis [30]. Furthermore, combining OCT quantitative data with clinical-pathological features can result in more precise tumor staging models, thereby improving the early diagnosis rate and treatment outcomes of UC [31].

#### 3.2.3 Case Studies and Clinical Validation

Several clinical studies have confirmed the efficacy of OCT in the early diagnosis of UC, yielding significant results. For instance, in a study by Bus *et al*. evaluating OCT as an *in vivo* staging and grading tool for upper tract urothelial carcinoma (UTUC), 26 patients underwent ureteroscopy (including biopsy and OCT scanning) followed by nephroureterectomy or segmental ureter resection. Standardized comparison showed that the lesion staging obtained by OCT matched the final histopathology in 83% of cases, with sensitivities and specificities for tumor invasion of 100% and 92%, respectively, validating OCT’s ability to visually observe, grade, and stage UTUC. Notably, Bus *et al*. also presented representative OCT cross-sectional images that intuitively depict different urothelial tumor phenotypes. In Ta lesions, the layered urothelial architecture remains recognizable; in invasive tumors, the normal stratification is completely effaced. In contrast, Carcinoma *In Situ* (CIS) is characterized by a thickened epithelial layer with reduced signal reflectivity. These typical images vividly underscore the intuitive advantage of OCT for visual staging and grading of UC [32].

In another Phase II multicenter clinical trial for real-time bladder cancer imaging and staging, OCT was used as an adjunct during cystoscopy. The results indicated that the prediction accuracy for invasive tumors was 84.7% with cystoscopy alone, compared to 91.7% with OCT-assisted cystoscopy (*P* = 0.063), demonstrating OCT's important supplementary role in intraoperative decision-making during transurethral resection of bladder tumors [33]. Additionally, clinical validation has shown that OCT has the potential for real-time monitoring of tumor changes, enabling physicians to dynamically assess tumor responses during treatment.

These studies not only validate the effectiveness of OCT in early diagnosis of UC but also lay the foundation for its broader adoption in urology.

### 3.3 Role of OCT in Intraoperative Monitoring and Guidance

#### 3.3.1 Enhancing Surgical Precision

As a high-resolution imaging technology, OCT provides real-time tissue structural information during surgery, which is crucial for improving surgical precision. OCT can image at the micron level, enabling surgeons to clearly observe minute changes in tissue structures, especially in ocular surgeries such as retinal and cataract surgeries [34, 35]. Research has shown that OCT can effectively monitor retinal status during surgery, ensuring both accuracy and safety. For example, during retinal detachment repair, OCT can assess the presence and changes in subretinal fluid in real-time, helping surgeons adjust surgical strategies to optimize outcomes [36]. Additionally, OCT helps surgeons evaluate the integrity of the internal membrane during surgery, ensuring that surrounding tissues are not damaged during membrane peeling, thus increasing the success rate of the procedure [37]. Furthermore, in radical prostatectomy for prostate cancer, nerve preservation is crucial for postoperative erectile function recovery, yet it remains a significant challenge. The main goal of the surgery is to preserve the continuity of the cavernous nerves that regulate erectile function [38]. Because the cavernous nerves are only a few hundred micrometers in diameter (typically 200–400 µm, and sometimes even smaller), they are difficult to identify and preserve with conventional visualization, making high-resolution modalities such as OCT attractive adjuncts for nerve-sparing prostatectomy [39, 40].

#### 3.3.2 Low-invasive Real-time Imaging

The low-invasive nature of OCT makes it an ideal choice for surgical monitoring. Unlike traditional imaging techniques, OCT does not require additional invasive procedures and can provide real-time tissue imaging without interfering with the surgery. This not only reduces patient discomfort but also lowers surgical risks. For instance, in ocular surgery, OCT can monitor the surgical area through a transparent optical window, providing essential visual information without disrupting the procedure [41]. In neurosurgery, OCT, with its non-invasive, high-resolution, and real-time imaging capabilities, can precisely detect deep brain hemodynamics, making it suitable for assessing conditions such as stroke, brain injury, and brain tumors [42]. This real-time imaging capability enables surgeons to promptly identify and address potential compli-cations, thus enhancing surgical safety and effectiveness [43].

#### 3.3.3 Intraoperative Monitoring and Surgical Guidance

Intraoperative OCT has already been widely adopted in other surgical specialties, such as ophthalmology and neurosurgery, where it is used for real-time tissue visualization and surgical guidance. These experiences demonstrate that OCT can be integrated into the operative workflow to provide high-resolution guidance without interrupting the procedure and offer conceptual lessons for its application in urologic surgery [44, 45]. In urologic oncology, intraoperative and perioperative OCT has been evaluated primarily as an adjunct to cystoscopy and endoscopy rather than as a standalone navigation tool. Prospective studies in bladder cancer have shown that integrating OCT with white-light cystoscopy during TURBT can improve real-time assessment of tumor presence and depth of invasion, with high sensitivity for malignant lesions and invasion beyond the lamina propria [46]. In the upper urinary tract, ureteroscopic OCT has been used intraoperatively to stage and grade UTUC and to distinguish non-invasive from invasive disease before definitive resection [32]. Pilot studies during nerve-sparing radical prostatectomy further suggest that OCT imaging of cavernous nerves and neurovascular bundles is technically feasible and may support real-time preservation of functional structures while maintaining oncologic control [47, 48].

### 3.4 Application of OCT in Treatment Outcome Evaluation for Urothelial Carcinoma

#### 3.4.1 Assessment of Tumor Residue and Recurrence

In recent years, OCT has demonstrated significant potential in evaluating treatment outcomes for UC. OCT provides real-time, micron-level tissue imaging, enabling physicians to accurately assess tumor boundaries and their relationship with surrounding tissues during surgery. Its advantage lies in its ability to monitor tumor residue in real time without the need for tissue biopsies [49]. By analyzing OCT images of surgical margins and surrounding lymph nodes, doctors can detect the presence of small amounts of tumor residue or lymph node metastasis, allowing them to promptly adjust subsequent treatment plans [50]. Additionally, OCT can be used to assess the risk of tumor recurrence. By observing postoperative OCT images, physicians can identify early signs of recurrence, such as abnormal angiogenesis or changes in tissue structure [51]. This ability to detect potential recurrence early enables timely intervention before tumor regrowth, improving patient prognosis.

#### 3.4.2 Real-time Monitoring of Treatment Effects

Another important application of OCT is the real-time monitoring of treatment effects in UC. For patients undergoing chemotherapy or radiation therapy, OCT can assist in evaluating the tumor's response. By regularly performing OCT imaging, physicians can observe changes in tumor volume, the recovery of tissue structure, and alterations in hemodynamics, all critical indicators of treatment efficacy. By comparing pre-treatment and post-treatment OCT images, doctors can quantify the extent of tumor shrinkage, determining the effectiveness of the treatment. This real-time feedback not only aids physicians in adjusting treatment plans but also enables more personalized treatment options for patients [4]. Moreover, OCT can be combined with other imaging techniques, such as MRI or CT scans, to enhance the evaluation of treatment outcomes. For instance, combining MRI or CT with OCT allows for a more comprehensive understanding of the overall treatment effect [52].

#### 3.4.3 Prognosis Evaluation

Prognosis evaluation is a key aspect of managing UC. The application of OCT in this area offers new perspectives on assessing patient prognosis. By analyzing tissue features in OCT images, doctors can identify biomarkers associated with prognosis. For example, OCT can reveal tumor microvascular density, cellular arrangement, and tissue heterogeneity, all of which are closely related to patient survival rates and recurrence risk [53]. Furthermore, certain OCT features, such as the clarity of tumor margins and the uniformity of internal structure, can effectively predict patient prognosis. Specifically, patients with unclear tumor margins or uneven internal structure typically have a poorer prognosis [54].

When considered along the entire UC management pathway, endoscopic OCT can contribute at several key decision points. During initial cystoscopic or ureteroscopic evaluation, OCT may assist in the early detection of flat or subtle lesions and in risk stratification by providing information on epithelial thickness and depth of invasion. In the operative setting, OCT can guide transurethral resection of bladder tumors and upper tract interventions by helping to delineate tumor margins and assess muscularis involvement in real time. After treatment, OCT has the potential to support post-treatment surveillance by facilitating the early detection of residual or recurrent disease in patients under cystoscopic or ureteroscopic follow-up. Taken together, these applications suggest that endoscopic OCT is best viewed as a complementary imaging modality that can add high-resolution structural information at multiple stages of UC care rather than as a stand-alone replacement for existing diagnostic tools.

Although many of the available studies report high sensitivities and specificities for endoscopic OCT in the detection and staging of UC, these findings should be interpreted with caution. Most published series are small single-center cohorts with selected patients, and negative or inconclusive studies are underrepresented in the literature. In combination with potential publication bias, this imbalance may lead to an overestimation of the diagnostic and staging performance of OCT relative to routine clinical practice. Larger, multicenter studies, including those that systematically report both positive and negative findings, are needed to more reliably define the true effectiveness of OCT.

### 3.5 Future Directions and Challenges

#### 3.5.1 Technological Innovations and System Upgrades

The application of OCT in UC is continually evolving to meet clinical needs and technological challenges. Future developments will focus on innovations and system upgrades to improve the accuracy and efficiency of OCT in UC detection. Although current OCT systems provide high-resolution images, there are still limitations in real-time imaging, depth penetration, and imaging speed. Additionally, noise and artifacts in OCT images can affect diagnostic accuracy, making the development of advanced image processing algorithms to enhance image quality a key research focus [55]. To address these issues, researchers are exploring new light sources, detectors, and image processing algorithms for faster and higher-quality imaging [56, 57].

Moreover, integration of Artificial Intelligence (AI) and Machine Learning (ML) with endoscopic OCT represents an important direction for future development. Early studies have shown that AI models trained on large OCT or clinical datasets can assist in more accurate identification of cancerous lesions and improve diagnostic sensitivity and specificity [58]. Beyond simple feature extraction, modern deep-learning approaches, such as convolutional neural networks and U-Net–like architectures, can be trained on OCT images to perform automatic layer segmentation, lesion detection, and assessment of depth of invasion. Applied to UC, such models could help distinguish benign from malignant lesions, predict muscularis invasion, and highlight suspicious areas in real time during cystoscopy or ureteroscopy, thereby supporting earlier diagnosis and more standardized, reproducible interpretation of OCT images [59].

However, the development of robust AI–OCT tools faces several challenges. Large, high-quality, and well-annotated multicenter OCT datasets are needed to avoid overfitting and to ensure that models generalize across different devices and institutions. Domain shift between centers, variations in acquisition protocols, and differences in image quality can all degrade model performance. Moreover, the limited interpretability of deep-learning models may hinder clinical acceptance, especially when AI-generated outputs appear to conflict with endoscopic findings. Future research should therefore focus on building multicenter OCT databases with standardized acquisition and annotation protocols, developing interpretable models that provide visual explanations or uncertainty estimates, and prospectively validating AI-assisted OCT workflows in UC against current guideline-based management strategies.

#### 3.5.2 Standardization and Normative Research

Currently, the application of OCT in UC lacks standardized protocols, leading to inconsistent results across hospitals and research institutions and affecting clinical decisions and patient management. Therefore, establishing standardized operational procedures and evaluation metrics will be a focus of future research [60, 61].

Standardization research should not only cover the technical aspects of imaging but also include data collection, processing, and analysis at every stage. For example, creating unified image acquisition protocols, standards for software usage, and formats for reporting results will improve reproducibility in research and the effectiveness of clinical applications [62]. At present, no dedicated international body has issued formal standards for urothelial OCT. Multicenter and international collaborations, including consortia and professional societies, will therefore be essential for developing consensus protocols, image-quality criteria, and reporting frameworks to promote standardization and global adoption.

#### 3.5.3 Clinical Promotion and Application Barriers

Despite the promising prospects of OCT technology in UC detection, several barriers still hinder its clinical promotion. Firstly, the acceptance and familiarity of clinical physicians with new technologies are key factors in their adoption. Many doctors may lack sufficient understanding of OCT's principles and applications, leading to reluctance in implementing the new technology in practice [63].

Secondly, the high cost of medical equipment is another significant obstacle. The procurement and maintenance costs of OCT systems can be high, limiting their use in certain hospitals, particularly in primary care settings. Encouraging the development of low-cost OCT systems could lower the entry barrier, allowing it to be applied in fields beyond UC [64, 65]. Additionally, healthcare insurance policies, including coverage scope and reimbursement standards, can impact the clinical application of OCT. To promote its adoption, relevant agencies need to collaborate with insurance companies to establish reasonable reimbursement policies to reduce the financial burden on patients [66].

Lastly, insufficient clinical research also limits the application of OCT technology in UC. Although preliminary studies have shown the potential of OCT in cancer detection, further large-scale clinical trials are needed to validate its effectiveness and safety [29]. Only with comprehensive clinical data will doctors and patients gain confidence in the technology, facilitating its widespread application in UC. As summarized in Fig. (**2**), the current development of endoscopic OCT for UC faces four major challenges, each of which is paired with potential solutions. Firstly, limited imaging depth restricts the ability of OCT to visualize deeper layers of the urinary tract wall and peritumoral tissue. Possible strategies to address this limitation include exploring new light sources with improved penetration, optimizing detector performance to enhance weak-signal capture, and developing more advanced reconstruction algorithms that can more accurately recover depth information from low-signal regions. Secondly, image noise and artifacts can compromise the reliability of layer segmentation and lesion detection. To mitigate this problem, dedicated noise-reduction algorithms can be designed to suppress speckle and system noise, the interferometric system can be optimized to reduce artifact generation, and overall system stability can be enhanced to minimize motion- and vibration-related interference. Thirdly, high equipment costs remain a barrier to widespread adoption. Potential solutions include researching and developing lower-cost light sources and optical components, simplifying detector and system designs to decrease manufacturing costs, and refining production processes so that OCT platforms can be produced and maintained at a lower price point without compromising image quality. Lastly, the current evidence base is limited by the small number and small sample sizes of clinical studies. To overcome this, multicenter clinical trials with larger sample sizes are needed, along with long-term follow-up studies to clarify the impact of OCT-guided strategies on patient outcomes. In addition, comparative studies that directly evaluate OCT against other diagnostic and intraoperative imaging modalities would help define the relative advantages, disadvantages, and appropriate indications for OCT in the management of UC.

In practice, the clinical translation of endoscopic OCT in UC could follow a staged approach. In the first phase, technical and procedural standardization is required, including consensus on image acquisition parameters, quality control, and reporting of OCT findings across centers. In the second phase, AI-assisted OCT interpretation, using deep-learning models trained on multicenter datasets for automated layer segmentation, lesion classification, and invasion depth estimation, should be integrated into clinical decision algorithms for non–muscle-invasive bladder cancer and UTUC and tested prospectively in real-time endoscopic workflows. In the third phase, comprehensive health-economic and implementation studies will be needed to evaluate capital and disposable costs, incremental diagnostic and staging benefits, cost-effectiveness, and the feasibility of incorporating OCT-based strategies into evidence-based guidelines and routine care pathways.

## CONCLUSION

Endoscopic optical coherence tomography provides high-resolution, real-time visualization of the urothelial microarchitecture. It shows considerable promise for improving the diagnosis, risk stratification, and treatment monitoring of UC throughout the urinary tract. Existing clinical studies indicate that endoscopic OCT can help differentiate benign from malignant lesions, estimate tumor stage by assessing depth of invasion, guide transurethral and ureteroscopic resections, and evaluate treatment response, thereby complementing conventional white-light imaging and cross-sectional modalities.

However, the current evidence base remains limited by small sample sizes, single-center designs, heterogeneous imaging and interpretation protocols, and relatively short follow-up durations. Furthermore, potential publication bias, where studies reporting positive or favorable OCT findings are more likely to be published than those with negative or inconclusive results, may lead to an overestimation of the apparent benefits of OCT and limit the completeness and generalizability of the available evidence. To move from promising early studies to routine clinical use, future research should prioritize multicenter prospective trials with standardized OCT acquisition and interpretation criteria and incorporate clinically meaningful endpoints such as progression-free and overall survival, organ preservation, and quality of life. Integrating OCT findings into decision algorithms for non–muscle-invasive bladder cancer and UTUC, and prospectively testing these algorithms against current guideline-based strategies, will be crucial to defining the specific indications and added value of OCT.

From a technological standpoint, further development is needed to extend imaging depth, reduce noise and artifacts, improve probe ergonomics, and facilitate seamless integration into endoscopic platforms and operating-room workflows. Parallel progress in artificial intelligence, including the development of robust, interpretable OCT-based models for automated layer segmentation, lesion classification, and real-time decision support, will require large, well-annotated multicenter OCT datasets and careful external validation. Health-economic analyses and implementation studies will also be important to clarify the cost-effectiveness and practical feasibility of broader adoption.

In summary, endoscopic OCT represents a powerful adjunct imaging modality with the potential to refine staging, guide surgical management, and ultimately improve long-term outcomes for patients with UC. Realizing this potential will depend on coordinated efforts in technological innovation, rigorous clinical validation, standardization, and integration of OCT into evidence-based care pathways.

## Figures and Tables

**Fig. (1):**
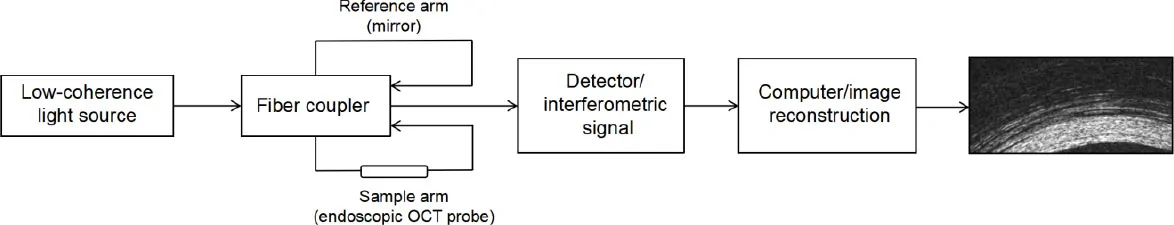
Basic working principle of endoscopic optical coherence tomography (OCT). Schematic of the endoscopic OCT system. Light from a low-coherence source is split by a fiber coupler into a reference arm and a sample arm. Light reflected from the reference mirror and backscattered from tissue at the tip of the endoscopic OCT probe recombines at the coupler to generate an interferometric signal, which is then detected and processed by the OCT engine to reconstruct depth-resolved cross-sectional images.

**Fig (2):**
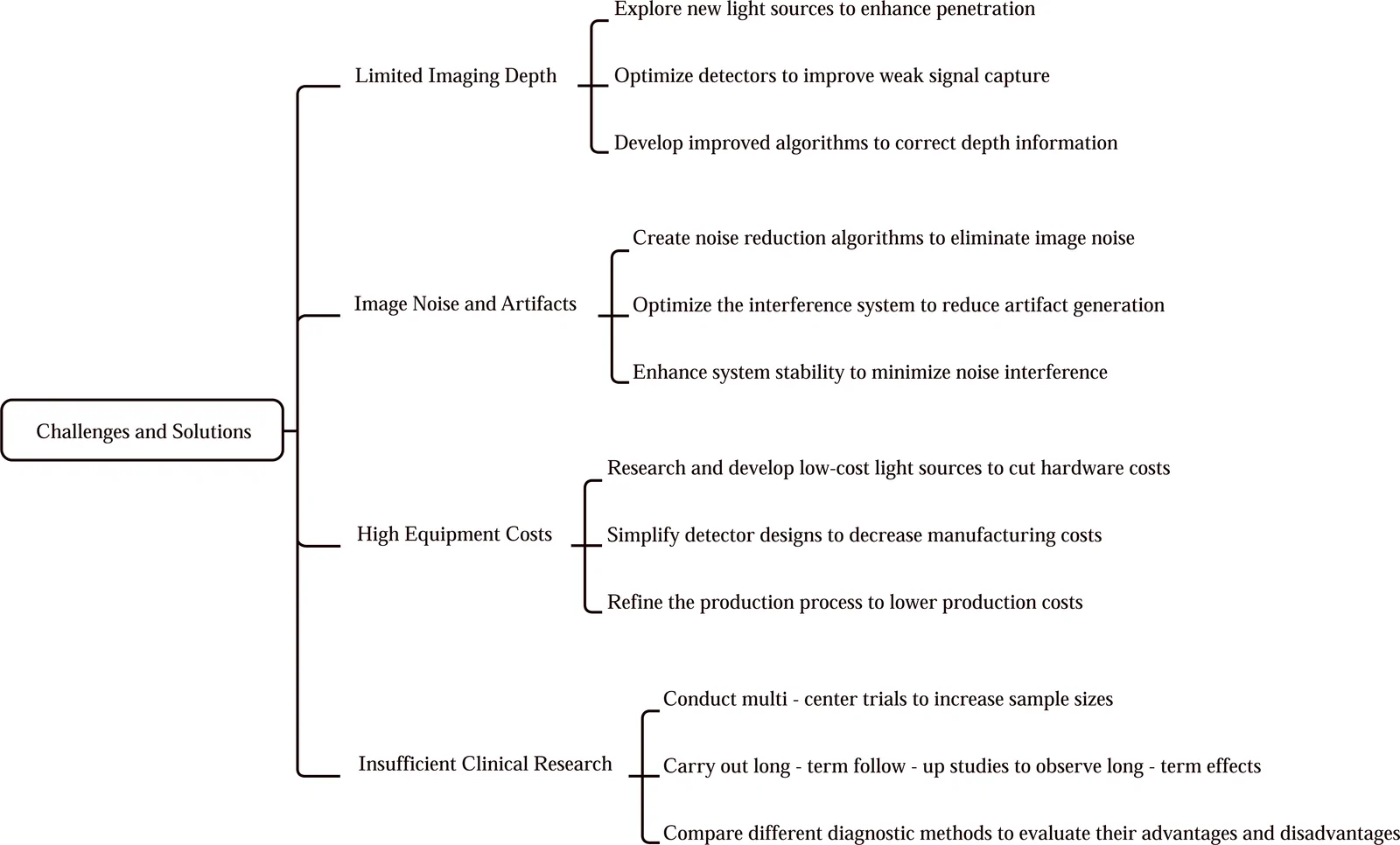
Challenges and potential solutions for the clinical translation of endoscopic OCT in urothelial carcinoma. The schematic summarizes four major challenges, such as limited imaging depth, image noise and artifacts, high equipment costs, and insufficient clinical research, and lists representative strategies to address each of them, including improvements in light sources and detectors, optimization of system design and stability, cost-oriented hardware simplification, and multicenter, long-term clinical studies.

**Table 1:** Comparison of OCT with other common medical imaging modalities.

** Imaging ** ** Modality **	** Spatial ** ** Resolution **	** Imaging ** ** Depth **	** Real-time ** ** Capability **	** Invasiveness **	** Clinical ** ** Applications **
Optical Coherence Tomography (OCT)	High (1–2 μm)	Shallow (2–3 mm)	High (real-time)	Non-invasive	Microscopic tissue analysis; suitable for superficial structures, e.g., urothelial carcinoma detection.
Computed Tomography (CT)	Low (>100 μm)	Deep (centimeter level)	Moderate	Minimally invasive (radiation exposure)	Whole-body imaging; useful for deep tissue evaluation and staging.
Magnetic Resonance Imaging (MRI)	Moderate (50–100μm)	Deep (centimeter level)	Low (longer scan time)	Non-invasive	Soft tissue imaging; commonly used for tumor staging and characterization.
Ultrasound (US)	Moderate (50–500 μm)	Deep (centimeter level)	High (real-time)	Non-invasive	Dynamic imaging; widely used for various organ assessments, including urinary system evaluation.

## LIST OF ABBREVIATIONS

AI: Artificial Intelligence
CLE: Confocal laser endomicroscopy
CT: Computed tomography
ML: Machine learning
MRI: Magnetic resonance imaging
NBI: Narrow-band imaging
OCT: Optical coherence tomography
PRISMA: Preferred Reporting Items for Systematic Reviews and Meta-Analyses
TURBT: Transurethral resection of bladder tumor
UC: Urothelial carcinoma
US: Ultrasound
UTUC: Upper tract urothelial carcinoma
